# The Influence of Social Media-like Cues on Visual Attention—An Eye-Tracking Study with Food Products

**DOI:** 10.3390/jemr18060062

**Published:** 2025-11-04

**Authors:** Maria Mamalikou, Konstantinos Gkatzionis, Malamatenia Panagiotou

**Affiliations:** Laboratory of Consumer and Sensory Perception of Food & Drinks, Department of Food Science and Nutrition, University of the Aegean, Metropolite Ioakeim 2, 81400 Myrina, Greece; fnsd20003@fns.aegean.gr (M.M.); teniapanag@aegean.gr (M.P.)

**Keywords:** marketing, endorser, social cues, social media, eye-tracking, attention, recall food evaluation

## Abstract

Social media has developed into a leading advertising platform, with Instagram likes serving as visual cues that may influence consumer perception and behavior. The present study investigated the effect of Instagram likes on visual attention, memory, and food evaluations focusing on traditional Greek food posts, using eye-tracking technology. The study assessed whether a higher number of likes increased attention to the food area, enhanced memory recall of food names, and influenced subjective ratings (liking, perceived tastiness, and intention to taste). The results demonstrated no significant differences in overall viewing time, memory performance, or evaluation ratings between high-like and low-like conditions. Although not statistically significant, descriptive trends suggested that posts with a higher number of likes tended to be evaluated more positively and the AOIs likes area showed a trend towards attracting more visual attention. The observed trends point to a possible subtle role of likes in user’s engagement with food posts, influencing how they process and evaluate such content. These findings add to the discussion about the effect of social media likes on information processing when individuals observe food pictures on social media.

## 1. Introduction

A rising number of companies rely extensively on social media as an effective channel to attract consumer attention to their products and consequently affect their purchasing decisions. Fundamental research on attention orienting indicate that humans selectively direct their focus in space, guided by either objectives or salient cues. Posner established a theory of attention that is based on target detection, visual orientation, and alertness [1]. On the other hand, according to Corbetta and Shulman [2], human visual attention is influenced by both top–down cognitive elements such as objectives, knowledge, and expectations as well as bottom–up factors such as sensory stimulation, demonstrating how perceptually salient stimuli naturally capture attention. Because consumers spend more time on digital media and less time on traditional media, advertisers and the marketing firms that represent them have to figure out how to successfully reach them on digital platforms [3]. In recent years, picture-based social media platforms such as Instagram have been extensively used by companies to promote their goods. Advertising efficacy starts by capturing audience attention, and every distinct advertising stimulus must first catch buyers’ attention, according to the paradigm proposed by Robert & Stahl [4]. Undoubtedly, promotional strategies can affect attention and enhance the efficiency of advertising in a number of ways.

In this context, one of the strategies that can differentiate and value items to attract the audience’s attention is the employment of the endorsement factor [5]. Endorsement, according to the Cambridge dictionary [6], is “any act of making a public statement of support for someone or something” (https://dictionary.cambridge.org/dictionary/english/endorsement, accessed on 15 January 2024). In this sense, social endorsement cues (SEC) that involve metric information about how other consumers have interacted or evaluated content on the internet, for example, the number of likes, shares, or comments on social media, assist individuals in making decisions about the value and trustworthiness of that content [7,8,9,10]. Likes are well known metrics in online and social media branding because they provide a numerical visual representation of social acceptance of products [11]. These symbolic cues reflecting consumers’ opinions, agreement, or attention in goods or brands symbolize electronic word-of-mouth eWOM [12,13,14]. eWOM communication can significantly impact information adoption, consumer beliefs, purchase intentions, loyalty to the brand, and trust [15,16,17]. In line with these, neurocognitive theories provide additional explanation of the influence of such cues. Falk, Way, & Jasinka [18] proposed the social reward/punishment framework that suggests two routes by which susceptibility to normative pressure happens. One route arises by the drive to pursue the social rewards conferred, and the second by the avoidance of social punishment, including social rejection. They suggest that generic variations that increase sensitivity to social cues may lead to greater conformity and susceptibility to normative social pressures, including rewards and punishments (or both). The combination of neurocognitive and behavioral perspectives indicates why likes go beyond just single symbolic signals and act like strong social reinforcements that direct attention and affect consumer behavior in digital contexts. Product likes differ substantially from product reviews and ratings. Product reviews and ratings have been shown to influence the choices of customers when assessing products [19,20]. They are often generated after consumers have purchased things and take more effort to create than product likes. Furthermore, product likes are only positive information through the aspect of the amount (number of product likes), whereas product reviews and ratings may convey positive, negative, or neutral information through the aspects of volume (number of reviews) and valence (number of star ratings). Research indicates that social signals such as likes might influence people’s views and behaviors towards products and brands. It is argued that the “like” signal encourages consumers to willingly support the advertiser and become one of their supporters [21]. The use of likes can impact consumer behavior in a number of ways. According to studies, posts with more likes gain more attention on social media networks [22,23,24]. Algorithms emphasize content with high engagement to appear in users’ feeds and recommendations [25,26,27]. One of the basic and pervasive types of social influence is conformity. Conformity is a social influence when individuals adjust their opinions or behaviors to fit in the group [28,29]. There are several adaptive advantages to fitting in with perceived social norms. People can enhance their precision, develop relationships, and maintain a positive self-concept by replicating the behavior of others [30]. The brain areas that generally appear to associate with conformity and responsiveness to social norms are the ventral striatum (VS) and ventromedial prefrontal cortex (VMPFC), which are essential for identifying salient cues and are also important parts of the brain’s reward system [31,32,33]. Neuroimaging research demonstrate that conformity serves motivationally important goals. This brain process explains why social cues such as likes are rewarding and increase adherence to information that is accepted by others. As more users are exposed to the information, its social validation can increase even more, increasing the opportunity for more likes and engagement.

Sherman and colleagues investigated teenagers’ reactions to photographs with social media likes on Instagram and found that teenagers were more inclined to press the like button on photos having more likes than fewer likes [34,35]. Borah et al. [36] examined the impact of Facebook likes on consumers’ opinions concerning health-related information, and revealed that social endorsement, in the form of Facebook likes, moderates the link between gain-framing message and source type on credibility choices. Likewise, Beukeboom et al. [37] found that Facebook likes have a favorable impact on customers’ brand assessments and purchase inclinations. This is because consumers see these likes as evidence of conversational human voice and interaction with the brands. Even when buyers do not share their thoughts on the Internet, the display of the amount of likes may implicitly influence their information reception and choice-making process [34,35,38,39,40]. Advertisements with a high number of likes, for example, were more likely to evoke engagement and were rated higher than those with less likes, particularly by frequent social media users [41].

Several recent studies have investigated the role of social networks and social endorsement in altering how people interact with food-related content [42,43,44]. Indeed, it has been stated that due to the prevalence of photos displaying unhealthy food and famous labels, a huge number of images shared on social media occur simultaneously with personal recommendations and may have a greater impact than advertising [45]. In a similar way Hawkins et al. [46] explored if socially endorsed images, like those posted on Instagram, influenced how many grapes and cookies people consumed. Whilst socially acceptable photos had no effect on participants’ individual consumption of grapes and cookies, viewing socially endorsed images of low energy density items (rather than healthy foods) resulted in people consuming greater amounts of grapes in grams than cookies. Recognizing and predicting human food preferences has long been a priority for sensory and consumer scientists. It is well known that several intrinsic as well as extrinsic factors can influence food choice, resulting in a potentially complicated array of variables [47]. Whereas first analyses depended on hedonic response data, implicit methods like eye-tracking have been increasingly relevant in consumer behavior research [48]. According to Stanton, Armstrong, and Huettel [49], neuromarketing is the application of neuroscience and physiological research approaches to acquire new insights into consumer behavior, preferences, decision-making, and other elements of human cognition and general behavior in relation to marketing. The eye-tracking method is extensively applied in cognitive psychology research. Numerous studies have to investigate consumers’ online behavior using eye-tracking. Luan et al. [50] utilized eye tracking to show that consumers allocate considerably more attention to feature-focused reviews compared to experiential reviews during product searches. The eye-tracking approach to investigating and interpreting consumer decision-making behavior and cognitive processing primarily relies on the eye–mind theory developed by Just and Carpenter [51]. According to Just and Carpenter [51], when a person looks, they are observing, considering, or attending to something, and eye movement can be used to identify cognitive processes. Eye movements are often employed as a measure of visual attention, as they can represent both covert and overt attention processes [52]. Consequently, eye-tracking techniques can provide novel insights into how consumers allocate their attention to visual stimuli such as print advertisements, websites, and packaging [53]. Eye-tracking applications monitor and analyze eye movements to assess visual attention [48]. While the technology does not provide insights automatically, it can be used in a variety of ways to investigate how attention is distributed across visual objects. Eye movements are particularly interesting when seeing commercials and, in general, because they provide fine-grained information on visual attention patterns. To see an object sharply, the image must be focused on the fovea, which spans roughly 2° of visual angle. This requires moving our eyes from one spot to the next, focusing on areas of interest [54,55]. Saccade eye movements are quick, well-coordinated adjustments that focus the fovea on visual stimuli. Their regulation is based on the integration of cognitive processes across various brain areas. Eye movements are therefore a crucial aspect of how we perceive and interact the visual environments. [56,57]. By examining the eye movement record, we can draw conclusions about how viewers selectively focus on the visual world, whether they are reading, viewing natural scenes, looking for a specific item, or, as is the primary interest here, watching advertising. As a result, understanding such trends can help improve marketing strategies and persuade prospective clients.

Consumer attention has been studied, and marketing scholars have proposed that it refers to the concentration of limited resources on a small amount of information [58]. Attention may determine the starting point of a consumer’s purchase behavior and influence their future decision-making, evaluation, and product selection throughout the shopping process [59]. Numerous studies have shown that eye movement, as a physiological response, can reflect an individual’s psychological activities and attitudes toward products [60,61]. Additionally, according to the food advertising hierarchy of effects paradigm [62], brand recall impacts attitudes and eating behaviors. After viewing advertisements, information may be stored in memory either explicitly or implicitly [63]. Enhanced cognitive processing can improve retention and recall [64], even though this depends on various factors such as individual differences, cognitive processes, attention, etc. [65].

As media use grows and multiple-device watching becomes the norm [66] (https://www.ofcom.org.uk/media-use-and-attitudes/media-habits-children/children-and-parents-media-use-and-attitudes-report-2019 accessed on 20 May 2024), just 10% of internet advertisements get viewed. Furthermore, the number of social media users in Greece at the start of 2024 was equivalent to 71.7 percent of the total population [67] (Digital 2024: Greece—DataReportal—Global Digital Insights, accessed on 20 May 2024). Therefore, it is crucial to understand what marketing content is being viewed on social media rather than just detecting its presence. Eye tracking is a commonly used indicator of attention selection [68] with longer and more fixations linked with a higher positive assessment of the item [69]. Social context is thought to play a key role in advertisement recall, awareness, and intent to buy [70] but evidence is limited, particularly in the social media context.

Given that most research indicates that viewing social media likes is connected with changes in consumer behavior and product sales [39,71], it is argued that product likes can serve as an informational cue, and consumers do consider the quantity of product likes when making decisions. Furthermore, people’s behavior changes based on the conclusions formed from the number of likes that products receive [11]. Because buyers associate likes with the sense that other consumers think positively about goods, the number of likes obtained by products may be used as a metric of social endorsement, indicating the products’ reputation among consumers [37]. As a result, products with a greater number of likes are regarded as more popular, which we believe leads to greater allocation of attention and recall. Given the fact that the amount of product likes may be a proxy for representing its popularity, we can assume that seeing a product with a higher number of likes may lead consumers to believe that the product is of different quality, resulting in increased attention paid to the product and better recall of it when compared to the same product with fewer likes. The present study aims to examine whether the number of likes on Instagram posts featuring traditional Greek foods influences attention, food recall, liking, perceived tastiness, and intention to taste. The study tested the following hypotheses: (H1) participants exposed to food images with a high number of likes will exhibit a longer total visit duration on the food items compared to participants exposed to the same images with a low number of likes; (H2) participants viewing images with a high number of likes will exhibit higher recall of the food items compared to those viewing the same images with fewer likes; (H3) areas of interest (AOIs) containing a high number of likes will attract a longer total visit duration than AOIs with low number of likes; and (H4) the number of Instagram likes will influence participants’ ratings of the food images such that food images with a high number of likes will be rated higher in liking, perceived tastiness, and intention to taste compared to the same images with a low number of likes. This study adds to the expanding literature of research on social media and eating behavior by investigating the effects of social endorsement (in the form of Instagram likes) on attention, memory, and food-related evaluations. This study highlights the potential of using social media cues to promote healthier and more traditional dietary choices, reduce health-related risks, and serve as an effective tool in food marketing strategies by identifying a potential mechanism through which consumer attention and decision-making can be influenced.

## 2. Materials and Methods

### 2.1. Samples and Methodology

A total of 99 volunteers ranging in age from 18 to 51 years (mean = 33.32 years) participated in this study. The number of participants was determined based on a comparable sample size employed in the related research by Mamalikou, Gkatzionis, & Panagiotou [42]. They were invited via email, telephone, or in-person communication. All had normal or corrected-to-normal vision, as assessed by self report. No formal screening for color vision deficiencies was conducted.

Being Instagram users was a prerequisite, operationalized as having an active Instagram account and engaging with the platform at least three times per week during the six-week period. The experimental procedures were ethically approved by the Department of Food Science and Nutrition at the University of the Aegean (protocol code-49262, date of approval 13 November 2024). The research was conducted between 20 November and 11 December 2024, over a period of three weeks. Informed consent was obtained from all participants involved in the study. The sessions were conducted in individual sensory booths at the Laboratory of Consumer and Sensory Perception of Food and Drinks of the University of the Aegean. The booths were closed sensory analysis cabins ensuring soundproofing. Informed consent was obtained from all participants. The participants were assigned to one of two conditions (control: low number of likes *n* = 49; experimental: high number of likes *n* = 50).

### 2.2. Design and Procedure

A between-subjects design was used, with one factor: socially endorsed images of two types: images of traditional Greek products socially endorsed with low number of likes and images of traditional Greek products socially endorsed with high number of likes. All participants were exposed to the same pictures, but under their group condition the image collection was ‘socially endorsed’ with a different (low vs. high) number of ‘likes’. The independent (manipulated) variable in this study is the number of Instagram likes presented in food-related Instagram images (high vs. low number of likes). The dependent variables include (a) visual attention directed to food area, (b) visual attention directed to the likes area, (c) memory of food names (free food recall), (d) subjective ratings of the food pictures in terms of liking, perceived tastiness, and intention to taste of food pictures.

The researchers employed an experimental design using eye-tracking as the main methodological tool to measure different levels of visual attention through variable manipulation. The study consisted of one session lasting approximately 20 min. A questionnaire was first given to all subjects to determine the sociodemographic characteristics of the research samples (gender, age) and we also collected several other variables to check as theoretical covariates. These variables included Instagram frequency of use, time spent on social media [72], usual traditional food intake, and level of hunger. Usual traditional food intake was recorded with a self-report frequency question asking participants “How often do you consume Greek traditional products?”; responses were given on a five-point Likert-type scale (1 = never, 5 = very often) and the level of hunger was measured with a Visual Analogue Scale (VAS) requesting participants to evaluate their level of hunger from 1 (not hungry at all) to 100 (extremely hungry). Participants were then asked to sit at approximately 40 cm to 60 cm from the monitor and to move as little as possible. The monitor was equipped with a Tobii Pro Spark eye-tracking camera (Tobii, Stockholm, Sweden) attached to its base (Tobii Pro Lab version 1.217.49450). The data were recorded and processed with the same software (Tobii Pro Lab version 1.217.49450), which was used to analyze gaze and pupil data and to calculate total visit duration. The system sampled gaze data at 60 Hz (default). Spatial precision—expressed as the root mean square (RMS) error—under ideal conditions is approximately 0.26°, and accuracy is approximately 0.45° of visual angle. The Tobii Pro Spark system has been validated by earlier research, which showed precise and trustworthy assessments of pupil and gaze data under comparable experimental circumstances on food-related behavior [73,74]. Eye-tracking cameras recorded eye movements that were analyzed to determine total visit duration (TVD) as an indicator of attention to the stimuli. TVD is the total amount of time the eye fixates on an area of interest (AOI) and is a relevant measure for analyzing attention. Areas of interest (AOI) are selected districts of a displayed stimulus. As mentioned by King, Cummins, & John (2019), TVD was found to be interrelated with decision-making and subjective attribute importance [75]. The areas of interest (AOIs) included in the study were food picture area, the main image of the post encompassing the visual content of interest, and the likes counter, encompassing the text and indicating the number of likes. AOIs boundaries were delineated in accordance with the research questions.

**Figure 1:**
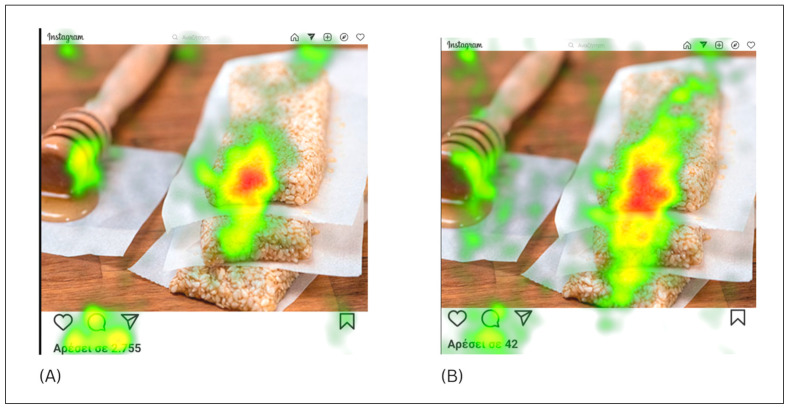
Example of heat map visualizations comparing participants visual attention to traditional food images with (**A**) high number of likes; (**B**) low number of likes.

The 99 participants were randomly assigned into two groups. The calibration and validation were performed before the start of the experiment. The first group (50 participants—experimental group 1) were exposed to the images socially endorsed with high number of likes (ranging from 2.575 to 3.819). The second group (49 participants—the control group 2) viewed the same pictures socially endorsed with a low number of likes (ranging from 27 to 85). Stimuli were 20 Instagram posts made for the task, containing 20 Greek traditional food images. Figure 1 represents one Greek traditional food image (Figure 1). The images were evaluated prior to the present study with a separate sample of participants (*n* = 50) to confirm that the images could be correctly identified as traditional by a significant majority of the participants. The depicted food was identified as traditional Greek food by 45 participants. The images were reproduced and presented as real Instagram posts. The technical specifications were as follows: (a) Image size: the images were displayed at 1080 × 1080 pixels. This corresponds to about 15 × 15 cm on a 17-inch screen with a resolution of 1920 × 1080. (b) Like-area size: the “like” area (counter) was presented at around 100 × 20 pixels (~1.96 × 0.39 cm). (c) Screen position: the figures were presented centrally on the screen. (d) A binocular (two-eye) calibration was performed at the start of each session, following a standard 5-point calibration protocol typically recommended for screen-based eye trackers. (e) Trial onset: each trial began with a fixation cross presented at the center of the screen (duration: ~500 ms), followed immediately by the stimulus image. Participants provided their VAS rating immediately after viewing each image. (f) Inter-trial interval: a blank screen (gray background) was then presented for 1000 ms before the next image appeared. The pictures were presented one at a time. Participants were asked to carefully observe all product pictures for exactly 13 s for each post. This time duration was based on pilot testing in order to verify that participants could observe the items successfully. Subsequently, participants were asked to rate each image on three Visual Analogue Scales (VAS; 0 = not at all, 100 = very much): (a) liking (“How much do you like the food shown in this image?”), (b) perceived tastiness (“How tasty do you think this food would be based on the image?”), and (c) intention to taste (“How much would you like to taste the food shown in the image?”). The use of Visual Analog Scales (VAS) was chosen over Likert-type scales due to their higher sensitivity and ability to capture subtle differences in responses. VAS has been widely used in sensory and consumer research to evaluate food-related perceptions due to its continuous nature and ease to use [76]. At the end of the session, participants were asked to freely recall and write down all the food names they recall having seen while viewing the Instagram images on a blank sheet of paper [77]. They were given 10 min to complete the task and the allocated time was the same for all participants.

## 3. Results

### 3.1. Eye-Tracking Measures of Attention

First of all, the baseline characteristics of the participants were analyzed by condition, to investigate whether they differed between the conditions. Results showed a significant main effect of condition for age (*p* < 0.001) between groups, with a mean age of 26 years (sd = 7.69 year) in the low likes group and mean age of 31 years (sd = 8.21 year) in high likes group. However, all participants were drawn from a relatively homogeneous population of young adults, and age-related effects on visual attention and related behaviors are expected to be minimal. Also, mean usual traditional food intake for low-likes group was M = 5.61 (sd = 1.19) and for high group was M = 5.78 (sd = 1.34), *p* = 0.23. Likewise, mean level of hunger for low-likes group was M = 39.16 (sd = 25.05) and high-likes group M = 35.8 (sd = 28.8), *p* = 0.44. Furthermore, mean number of Instagram accounts followed in low-likes group was M = 717.04 (sd = 711.38) and high-likes M = 547.67 (sd = 530.53), *p* = 0.12. Sample descriptive statistics characteristics for continuous variables are in Table 1. There were no other significant differences between the groups.

To check if there were differences in the visual attention between food pictures with high number of likes and pictures with low number of likes, we ran independent *t*-tests with the visual attention metric of TVD in seconds (total visit duration) of AOI–food area for all 20 pictures. Raw *p*-values are reported and significance determined using Bonferroni correction with α = 0.0025. The results revealed that there were no significant differences in visual attention of food area between groups (see Appendix A Table A1) for all 20 food pictures. Particularly, mean total visit duration for high number likes of food area was M = 10.35 s (sd = 1.9) and mean total visit duration for low number likes was M = 10.32 (sd = 1.99). Likewise independent *t*-tests were conducted to check if there were differences in visual attention metric of TVD of AOI–likes area. Raw *p*-values are reported and significance determined using Bonferroni correction with α = 0.0025. The results revealed that participants paid significant more attention to AOI with high number of likes in 4 of 20 pictures (Table 2). More specifically mean total duration for high-likes area was M = 0.24 (sd = 0.77) and mean total visit duration for low-likes area was M = 0.11 (sd = 0.31). Τhe descriptive statistics revealed consistent directional trends where mean viewing times were consistently higher for high-likes AOIs. Additionally in the heat map pictures it is indicated that AOI with high number of likes shows a redder and more extended viewing zone than the AOI with low number of likes, whereas the AOI–food area with a low number of likes shows redder and more focused viewing pattern of the food than the AOI with high number of likes. However, the heat maps are presented for illustrative purposes only (see Figure 1 and Supplementary Figure S1), while statistical comparisons are based on the inferential analyses.

### 3.2. Food Name Recall

A factorial ANOVA was conducted to compare the effects of the group (high number of likes versus low number of likes) on free food name recall. The results revealed that there were no statistically significant differences between groups F(1.88) = 1.23, *p* = 0.27 in food name free recall indicating no significant difference in food name recall between groups. Participants in the group with the high number of likes recalled on average 53% of the foods and in the group of low likes 54.34% of the food names.

### 3.3. Liking, Perceived Tastiness, and Intention to Taste

To examine whether the number of Instagram likes influenced subjective evaluations of food pictures a series of MANCOVAs were conducted, one for each of the 20 food images. In each analysis, the number of likes (high vs. low) served as the independent variable, while liking, perceived tastiness, and intention to taste were entered as dependent variables. Raw *p*-values are reported and significance determined using Bonferroni correction with α = 0.0025. (See Appendix A Table A2). Age and social media usage were included as covariates to control for their potential effects. The multivariate analysis revealed that there were no significant differences in liking, perceived tastiness and intention to taste between groups. However, inspection of group means (see Appendix A Table A2) suggested consistent trends across conditions. Specifically, out of the 20 food images: in 13 images, the high-like condition resulted in higher means ratings for liking. In 14 images, higher means were observed for high-likes condition for perceived tastiness, and finally, in 15 images participants reported greater intention to taste compared to those in low-like condition. These findings suggest a potential directional effect of social endorsement (in the form of Instagram likes) on subjective food evaluations, even in the absence of statistically significant differences. The pattern suggests that a higher number of likes may positively bias users’ perceptions and behavioral intentions regarding food products on social media platforms.

## 4. Discussion

There has been extensive research on the various social factors influencing human eating behavior, relatively little attention has been given to the impact of social media cues—particularly ‘likes’—on individuals’ attention, memory, liking, perceived tastiness, and intention to consume food products, especially traditional Greek food. To our knowledge, this paper is a pioneer in empirical investigations examining the effectiveness of social media likes as implicit endorsements of Greek traditional food posts, focusing on their influence on visual attention, memory, and evaluative responses using eye-tracking technology. The aim of this study was to investigate how much attention is allocated to food posts on social media that are endorsed with likes, and the extent to which their content is remembered, liked, perceived as tasty, and elicits intention to taste. We hypothesized that participants would exhibit greater visual attention toward Instagram food posts endorsed with higher number of likes—interpreted as social approval—compared to posts with a lower number of likes. Consequently, we anticipated that food posts with more likes would lead to enhanced memory of food names, greater liking, increased perceived tastiness, and a stronger intention to taste the food presented.

Our findings provide nuanced insights into how social media cues, particularly Instagram likes, influence users’ attention, memory, and evaluation of traditional Greek food posts. Contrary to our initial hypothesis, participants did not exhibit significantly greater overall visual attention to food posts, as indicated by the nearly identical total visit durations for high like (M = 10.35 s) and low like (M = 10.32 s). In a similar manner, statistically significant differences were observed only in 4 out of 20 images, with participants allocated significantly more visual attention to AOIs with a high number of likes compared to low-likes areas. Nevertheless, the descriptive statistics revealed consistent directional trends where mean viewing times were consistently higher for high-likes AOIs (mean low likes area = 0.11; mean high likes area = 0.24) (see Table A2). Additionally, in the heat map pictures we observe that AOI with high number of likes shows a redder and more extended viewing zone than the AOI with low number of likes, whereas the AOI–food area with a low number of likes shows redder and more focused viewing pattern of the food than the AOI with high number of likes. This suggests that the number of likes influenced where participants looked within the image rather than the overall amount of visual attention. The heat maps are presented for illustrative purposes only, while statistical comparisons are based on the inferential analyses. This pattern may indicate a systematic bias in attentiveness to social acceptance cues. This selective attention can be explained by the social proof paradigm, which holds that people use signals of group acceptability, such as like counts, as social validation heuristics [78].

In digital environments, such cues have been shown to disproportionately direct attention and affect engagement behaviors [79,80]. Eye-tracking evidence suggests that social endorsement indicators serve as prominent stimuli, grabbing early visual attention [81]. Regarding memory performance, the analysis revealed no significant differences between conditions. Participants in the high-like condition recalled 53% of food names while those in the low-like group recalled 54.34%, indicating no memory enhancement linked to social endorsement. Similarly, the multivariate analysis of subjective evaluations revealed no significant differences between groups. These results may be explained by baseline familiarity with traditional products, meaning that participants had already been in direct, real, and personal contact with the stimulus, therefore likes had a secondary impact on their evaluations. Nevertheless, group mean comparison revealed consistent directional trends: in 13 out of 20 images, higher likes led to greater liking; in 14 images, higher perceived tastiness; and in 15 out of 20 images stronger intention to taste. Taken together, these findings suggest that while Instagram likes may not significantly enhance attention or memory for food content, they subtly shape evaluative judgments, potentially biasing users toward more positive perceptions and intentions when posts are socially endorsed. This points to a psychological mechanism through which social media engagement—via visible approval signals like likes—may influence consumer behavior even in the absence of overt attention or cognitive effects.

The above-mentioned findings suggest that Instagram likes as a form of social endorsement, in posts with traditional Greek food, may operate in a more implicit manner among social media groups and consequently have limited influence on human eating behavior, as there was no clear mention of the norm (likes) to the participants and participants did not explicitly state the number of likes or hypothesized on the purpose of the study. Although no statistically significant differences emerged, the descriptive analysis revealed a consisted trend in which high-like AOIs attracted more mean visual attention, a pattern further supported by the heat map visualizations. Instead, the large quantity of AOIs may have served primarily as visual salient cues that immediately drew attention rather than being processed as useful social information. In line with this, Itti and Koch created a computational model of attention [82] in which the visual system combines two channels—responses for color, brightness intensity, and orientation—to produce a saliency map. In this concept, the most salient area in an image is the one holding the most significant information, attracting the focus of attention, which is a small disk reflecting the section of the image an observer focuses or fixates on. According to this viewpoint, likes may not function primarily as social endorsement signals, but rather as visually significant items that automatically draw attention within the photos. Consequently, participants would have been less able to adjust their behavior to be in line with the provided norm if they were unaware of it [83].

These findings align with prior work to suggest that a high number of ‘likes’ decreased the attention given to news posts [84] nor do they predict participants’ HED (high energy dense) and LED (low energy dense) food consumption [46]. Similarly, in a study with 149 participants, Mamalikou, Gkatzionis, & Panagiotou revealed that Instagram likes as a form of social endorsement cues in traditional Greek food Instagram posts did not incite people to consume more of either traditional rusks or modern crackers [42]. Meta analytic evidence by Haim et al. [70] further indicates that popularity cues exhibit inconsistent impacts on selection and behavioral measures. This could be due to insufficient visual attention to the cues. Further, an eye-tracking experiment with university students revealed that social media users tend to fixate longer on textual post elements like headlines than other post elements like pictures or likes [85], probably because they need fewer cognitive recourses and shorter time to process than text [86]. Additionally, research has examined the impact of food image content (healthy vs. unhealthy) on digital engagement. The findings indicated that when a person is pictured next to a healthy food post, participants’ likelihood to try the product and engage with it (such as like it) are higher [43]. The findings from the experiment reveal that the underlining process leading to this effect may be the possibility of identification with the person in the image (influencer) which in alignment suggests that posts featuring human faces are 38% more likely to receive likes and 32% more likely to receive comments. This implies that social media posts featuring human elements receive more attention and engagement [87]. In line with the food image content, Murphy et al. [77] showed that advertisements of unhealthy food elicited significantly more positive responses than non-food and healthy food: participants were more likely to want to ‘share’ unhealthy posts; rated peers more positively when they had unhealthy posts in their feeds; remembered and recognized a greater number of unhealthy food brands; and viewed unhealthy advertising posts for longer periods of time. Traditional Greek dishes are made with healthy ingredients and make for good nutrition choices. However, hashtag usage data for #GreekFood (~46,337) [88] and #greecefood (~24,338) [89] show low social media sharing. As a result, even when traditional Greek food photos were endorsed with social endorsement cues (likes), they did not receive the expected attention emphasis.

Furthermore, according to the levels of processing theory introduced by Craik and Lockheart [90], shallow processing of information, such as simply watching the number of likes on social media, results in a poor memory retention compared to a more intense engagement such as commenting or reading [91]. Consistent with the Self-Reference Effect (SRE) proposed by Rogers, Kuiper, & Kirker [92] which suggests that people better remember and process information that is relevant to themselves; as a result, even if a food-related social media content is socially endorsed with high number of likes, it may not be memorable if it is not personalized to the viewers’ personal interests and needs. The above-mentioned studies demonstrate that although likes represent a form of social validation which can possibly attract more attention, the context and content of social media posts are also important in influencing where people tend to look during social media use. Finally, there are users who observe these metrics with skepticism, recognizing their boundaries and potential for manipulation [93].

The findings suggest that the ways in which social media norms, in the form of Instagram likes, influence attention, memory, and subjective evaluations of food posts are still not fully understood, making it difficult to determine how and why eating-related content affects food-eating behavior. As with all research, the findings of this study must be interpreted considering substantial limitations. Firstly, the eye-tracking experiment was conducted in a laboratory. Although there was no effect of Instagram likes on attention, memory, and subjective evaluations of food in the laboratory setting where artificial Instagram posts were shown to the participants, it is unclear whether this conclusion translates to real-life social media use. Second, the image order was purposely maintained to guarantee that all participants saw the stimuli in the same order, allowing for paired comparisons between conditions. Third, visual saliency was not strictly regulated because high-like-count posts differed from low-like-count ones in terms of character length. Stimuli were delivered in their natural form to maintain ecological validity. Finally, the food images were not standardized in terms of luminance or chromaticity. Even though color can influence visual attention, this was beyond the scope of the current study. Despite these limitations, this study is one of the few that examines social norms, for instance Instagram like cues, communicated via social media and their impact on attention, recall, and subjective evaluations regarding traditional food products with the use of eye-tracking technology.

## 5. Conclusions

In conclusion, the present study indicates that Instagram likes have a limited effect on users’ visual attention, memory, and evaluative responses towards traditional Greek food posts. Although not statistically significant, descriptive trends suggested that posts with a higher number of likes tended to be evaluated more positively and the AOI corresponding to the likes area showed a tendency to attract more visual attention. These trends point to the possible subtle role of likes. Consequently, relying solely on social media likes, as a marketing strategy for traditional food products, may not be an efficient method of capturing consumer attention or enhancing memorability of food items. Marketers should therefore combine Instagram likes with additional marketing strategies, such as collaboration with influencers, visually appealing content, or digital storytelling. Future research could build upon this study by exploring the impact of Instagram likes on consumer’s actual purchase intentions or eating behavior. Moreover, it could investigate the effects of likes on short-form social media platforms such as TikTok (v12.3.7.1), as well as their influence across different food and non-food product categories, and among participants with diverse characteristics, such as adolescents and Generation Z.

## Figures and Tables

**Table 1:** Sample descriptive statistics for all participants, split by condition.

			Group		
		Low Number Likes		High Number Likes	
		Ν	%	Ν	%
Gender	Male	15	30.60	11	22.00
	Female	34	69.40	39	78.00
Time on Instagram	<10 min	5	10.20	6	12.00
	10–30 min	5	10.20	7	14.00
	31–60 min	16	32.70	17	34.00
	>60 min	23	46.90	20	40.00
Picture uploads	Never	1	2.00	2	4.00
	<1/month	38	77.60	36	72.00
	2–3 month	3	6.10	3	6.00
	1 week	3	6.10	1	2.00
	2–3 week	4	8.20	6	12.00
	Daily	0	0.00	2	4.00
Picture content	Family, friends, famous	1	2.00	0	0.00
	Other content/objects	1	2.00	1	2.00
	Animals	0	0.00	0	0.00
	Μemes/quotes	0	0.00	3	6.00
	Landscapes/locations	11	22.40	15	30.00
	Food	1	2.00	1	2.00
	Selfies	7	14.30	9	18.00
	Pictures with you and your friends	28	57.10	21	42.00

**Table 2:** Total visit duration of AOI likes area.

	Total		Low Number Likes		High Number Likes		
	Mean	SD	Mean	SD	Mean	SD	*p*
likes area 1	0.39	0.61	0.20	0.31	0.58	0.77	0.00 *
likes area 2	0.33	0.86	0.27	1.05	0.39	0.63	0.01
likes area 3	0.20	0.32	0.09	0.15	0.30	0.41	0.001 *
likes area 4	0.16	0.31	0.10	0.22	0.23	0.37	0.02
likes area 5	0.24	0.46	0.16	0.40	0.31	0.51	0.02
likes area 6	0.15	0.33	0.06	0.12	0.24	0.43	0.00 *
likes area 7	0.13	0.29	0.11	0.35	0.15	0.22	0.04
likes area 8	0.10	0.20	0.08	0.18	0.12	0.21	0.36
likes area 9	0.15	0.25	0.10	0.21	0.20	0.28	0.06
likes area 10	0.23	0.62	0.15	0.34	0.30	0.80	0.18
likes area 11	0.15	0.27	0.13	0.26	0.18	0.28	0.27
likes area 12	0.19	0.38	0.12	0.31	0.26	0.43	0.04
likes area 13	0.13	0.24	0.06	0.17	0.19	0.29	0.00 *
likes area 14	0.22	0.42	0.13	0.20	0.31	0.54	0.04
likes area 15	0.12	0.24	0.07	0.14	0.18	0.30	0.03
likes area 16	0.13	0.22	0.11	0.19	0.14	0.25	0.52
likes area 17	0.13	0.32	0.07	0.20	0.19	0.40	0.06
likes area 18	0.14	0.31	0.09	0.25	0.20	0.35	0.03
likes area 19	0.12	0.20	0.08	0.15	0.16	0.23	0.03
likes area 20	0.13	0.31	0.05	0.10	0.21	0.41	0.01

Note: AOI: areas of interest, SD: standard deviation; *p*-values derived from the *t*-test. The Bonferroni corrected significance level was set at 0.0025. * Denotes statistically significant results (*p* ≤ 0.0025).

## Data Availability

The original contributions presented in this study are included in the article and Supplementary Materials. Further inquiries can be directed to the corresponding author upon request.

## Supplementary Materials

The following supporting information can be downloaded at: https://www.mdpi.com/article/10.3390/jemr18060062/s1, Figure S1: Heat map visualizations comparing participants visual attention to traditional food images: (A) high number of likes; (B) low number of likes.

## Appendix A

**Table A1:** Total visit duration of AOI food area.

	Total		Low Number Likes		High Number Likes		
	Mean	SD	Mean	SD	Mean	SD	*p*
food area 1	9.76	1.93	9.71	1.99	9.81	1.90	0.824
food area 2	9.95	2.12	10.07	2.09	9.84	2.17	0.641
food area 3	10.02	1.92	10.05	1.52	9.99	2.26	0.795
food area 4	10.36	2.10	10.76	1.46	9.99	2.53	0.186
food area 5	9.76	2.25	9.67	2.04	9.85	2.45	0.438
food area 6	10.42	1.77	10.55	1.55	10.30	1.97	0.579
food area 7	10.49	1.78	10.66	1.49	10.33	2.02	0.561
food area 8	10.47	1.77	10.39	1.79	10.55	1.76	0.577
food area 9	10.21	2.26	10.32	2.06	10.10	2.45	0.805
food area 10	10.10	2.07	10.12	2.05	10.09	2.11	0.941
food area 11	10.24	2.18	9.95	2.50	10.51	1.80	0.282
food area 12	10.43	2.05	10.29	2.01	10.56	2.10	0.370
food area 13	10.76	1.30	10.74	1.41	10.78	1.19	0.885
food area 14	10.41	1.87	10.46	1.70	10.36	2.04	0.977
food area 15	10.44	1.69	10.13	1.95	10.74	1.36	0.079
food area 16	10.51	1.77	10.11	2.20	10.89	1.13	0.042
food area 17	10.73	1.78	10.70	1.65	10.75	1.91	0.614
food area 18	10.84	1.46	10.98	1.19	10.71	1.69	0.480
food area 19	10.26	2.35	10.05	2.51	10.46	2.19	0.326
food area 20	10.58	1.78	10.68	1.44	10.48	2.07	0.761

Note: AOI: areas of interest, SD: standard deviation; *p*-values derived from the *t*-test. The Bonferroni corrected significance level was set at 0.0025.

**Table A2:** Means, standard deviations, and *p*-values for liking, perceived tastiness, and intention to taste across 20 food images, by Instagram like condition.

	Low Number Likes		High Number Likes		
	Μean	SD	Μean	SD	*p*
Image 1 Liking	59.80	21.65	60.2	27.61	0.729
Image 1 Perceived Tastiness	65.31	19.85	62.7	27.03	0.933
Image 1 Intention to Taste	63.98	28.04	62.1	34.51	0.925
Image 2 Liking	43.67	30.24	50.4	30.10	0.002
Image 2 Perceived Tastiness	44.80	30.65	49.9	30.19	0.012
Image 2 Intention to Taste	44.80	33.38	55.0	31.98	0.013
Image 3 Liking	58.98	29.84	59.8	30.14	0.216
Image 3 Perceived Tastiness	58.47	29.71	63.1	27.33	0.164
Image 3 Intention to Taste	60.61	33.97	62.9	31.27	0.116
Image 4 Liking	52.45	33.67	64.9	27.40	0.256
Image 4 Perceived Tastiness	53.37	34.95	67.7	28.18	0.465
Image 4 Intention to Taste	50.61	36.62	65.4	29.69	0.462
Image 5 Liking	64.80	27.50	69.6	24.76	0.175
Image 5 Perceived Tastiness	66.84	29.13	74.0	22.72	0.271
Image 5 Intention to Taste	67.24	30.36	72.2	25.26	0.634
Image 6 Liking	71.94	28.59	70.8	30.04	0.875
Image 6 Perceived Tastiness	78.47	20.39	71.6	29.65	0.765
Image 6 Intention to Taste	69.49	31.25	71.1	31.82	0.723
Image 7 Liking	79.59	22.45	82.2	20.73	0.353
Image 7 Perceived Tastiness	81.63	21.25	84.1	17.72	0.679
Image 7 Intention to Taste	79.80	24.24	85.8	20.69	0.888
Image 8 Liking	78.57	22.68	72.1	25.24	0.586
Image 8 Perceived Tastiness	81.22	20.90	75.9	23.68	0.698
Image 8 Intention to Taste	74.08	27.68	67.5	30.26	0.672
Image 9 Liking	74.69	27.45	79.2	23.00	0.725
Image 9 Perceived Tastiness	77.65	27.14	81.3	23.12	0.815
Image 9 Intention to Taste	75.10	31.71	78.2	28.51	0.706
Image 10 Liking	85.10	18.58	83.0	18.15	0.266
Image 10 Perceived Tastiness	85.82	18.72	85.9	16.77	0.738
Image 10 Intention to Taste	88.16	20.15	84.9	18.14	0.962
Image 11 Liking	77.45	19.18	82.4	19.70	0.953
Image 11 Perceived Tastiness	78.78	17.63	83.1	18.79	0.846
Image 11 Intention to Taste	75.61	21.35	77.2	25.93	0.576
Image 12 Liking	55.20	25.66	62.9	30.54	0.970
Image 12 Perceived Tastiness	60.10	23.11	64.2	32.89	0.870
Image 12 Intention to Taste	53.67	28.48	58.2	34.12	0.596
Image 13 Liking	65.71	27.58	62.5	32.78	0.071
Image 13 Perceived Tastiness	65.10	28.46	66.8	30.60	0.504
Image 13 Intention to Taste	56.63	30.51	63.4	35.69	0.431
Image 14 Liking	74.80	26.89	69.0	31.30	0.803
Image 14 Perceived Tastiness	75.61	27.00	71.1	32.69	0.806
Image 14 Intention to Taste	65.41	32.69	67.7	32.34	0.584
Image 15 Liking	82.24	21.77	73.3	26.30	0.338
Image 15 Perceived Tastiness	83.16	22.56	77.1	23.99	0.370
Image 15 Intention to Taste	81.22	24.25	70.0	31.61	0.696
Image 16 Liking	55.92	32.77	58.4	32.68	0.016
Image 16 Perceived Tastiness	57.14	30.41	63.6	30.89	0.009
Image 16 Intention to Taste	46.22	35.67	51.2	34.59	0.033
Image 17 Liking	86.53	14.30	86.2	19.78	0.155
Image 17 Perceived Tastiness	89.29	14.43	88.6	18.93	0.348
Image 17 Intention to Taste	90.10	16.09	87.9	20.28	0.758
Image 18 Liking	51.02	34.57	56.5	28.74	0.565
Image 18 Perceived Tastiness	48.06	34.52	56.0	29.24	0.973
Image 18 Intention to Taste	42.35	35.94	52.3	32.25	0.583
Image 19 Liking	65.31	30.80	74.8	27.65	0.886
Image 19 Perceived Tastiness	70.82	29.76	78.7	25.29	0.765
Image 19 Intention to Taste	55.82	34.89	65.3	31.36	0.450
Image 20 Liking	56.33	32.26	59.2	30.89	0.497
Image 20 Perceived Tastiness	57.96	32.88	62.4	31.87	0.053
Image 20 Intention to Taste	45.71	36.14	52.7	31.90	0.062

Note: LN likes: low number likes; reported *p*-values correspond to the test between subject effects from the MANCOVA; the Bonferroni corrected significance level was set at 0.0025.
