# Supplementary material for: The Influence of Social Media-like Cues on Visual Attention—An Eye-Tracking Study with Food Products
Source: J Eye Mov Res. 2025 Nov 4;18(6):62. doi: 10.3390/jemr18060062 (PMC12641725; doi:10.3390/jemr18060062)

Fig. S1 Heat map visualizations comparing participants visual attention to traditional food images: (A) high number of likes; (B) low number of likes.

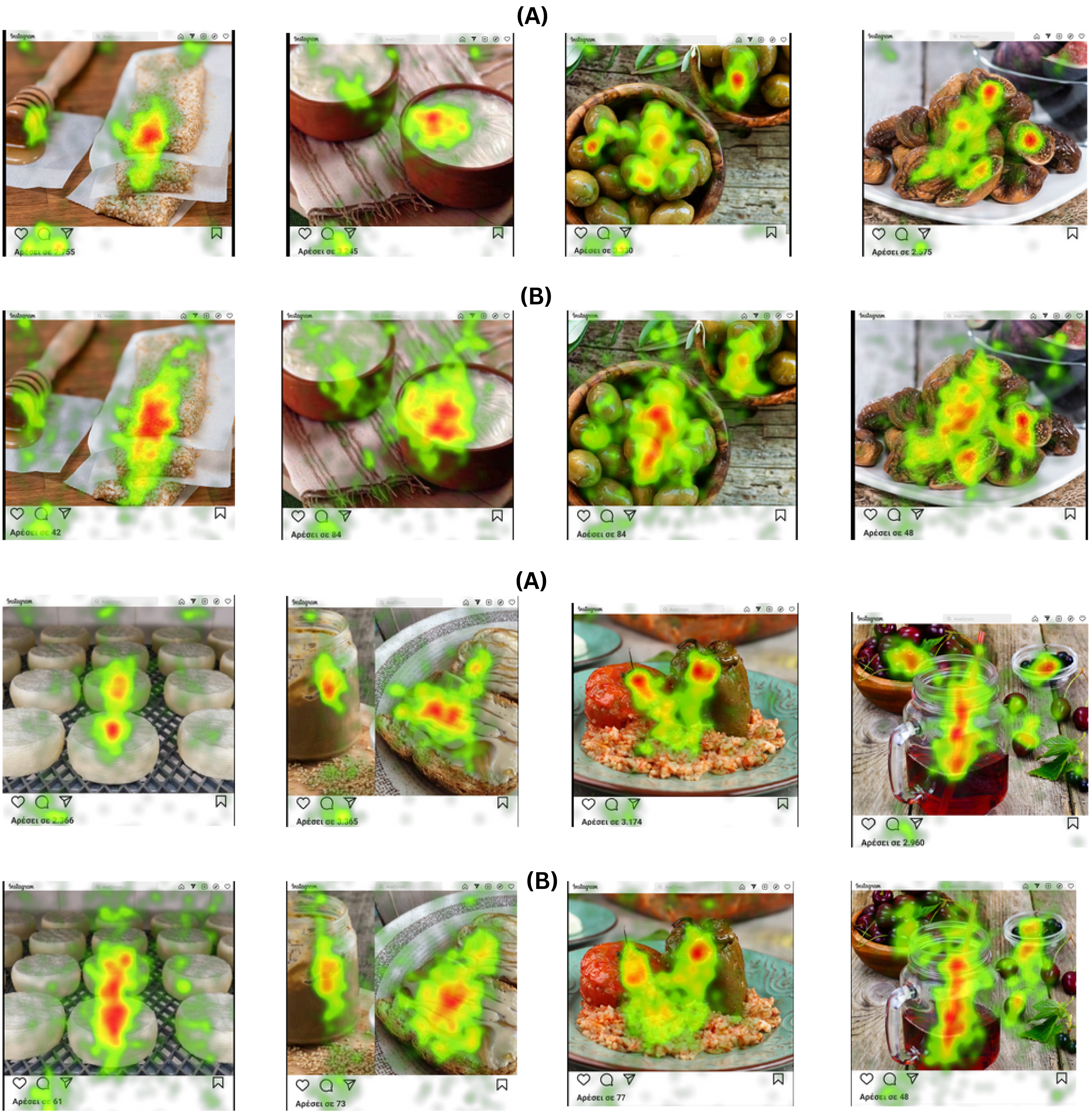

Heat map visualizations comparing participants visual attention to traditional food images: (A) high number of likes; (B) low number of likes.

(A)

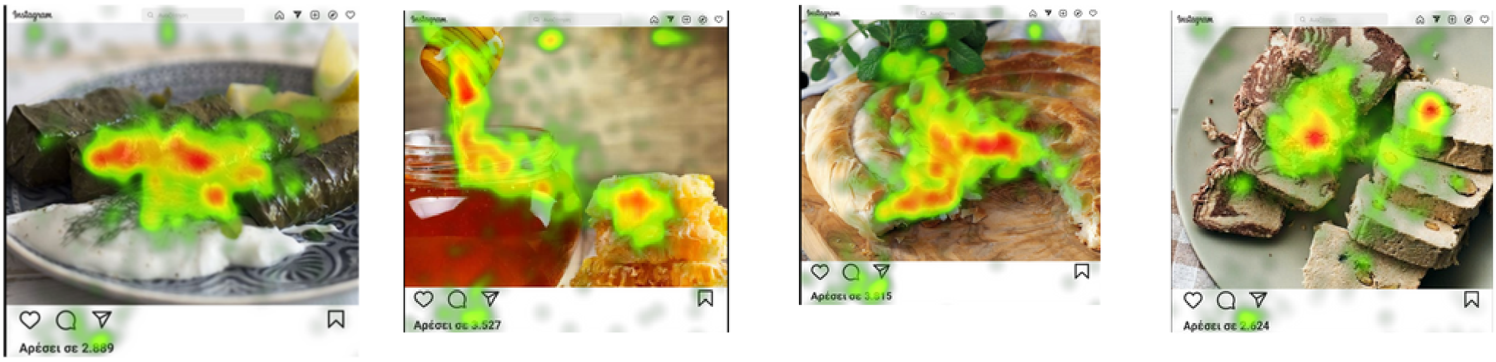

(B)

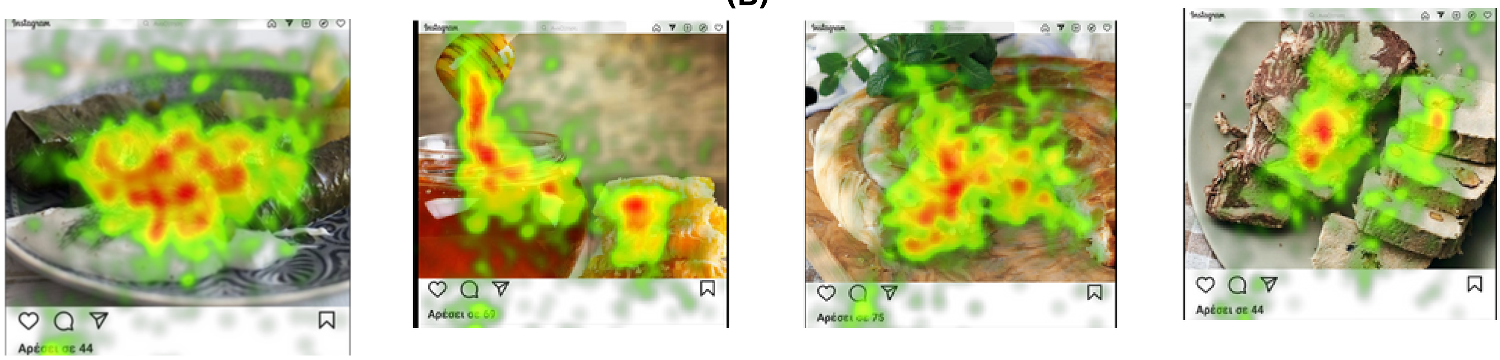

(A)

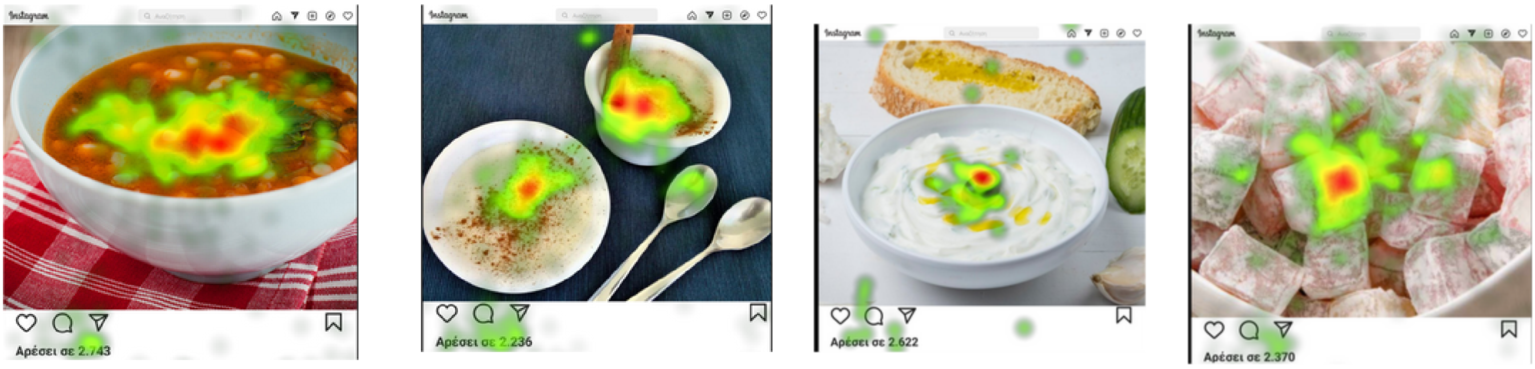

(B)

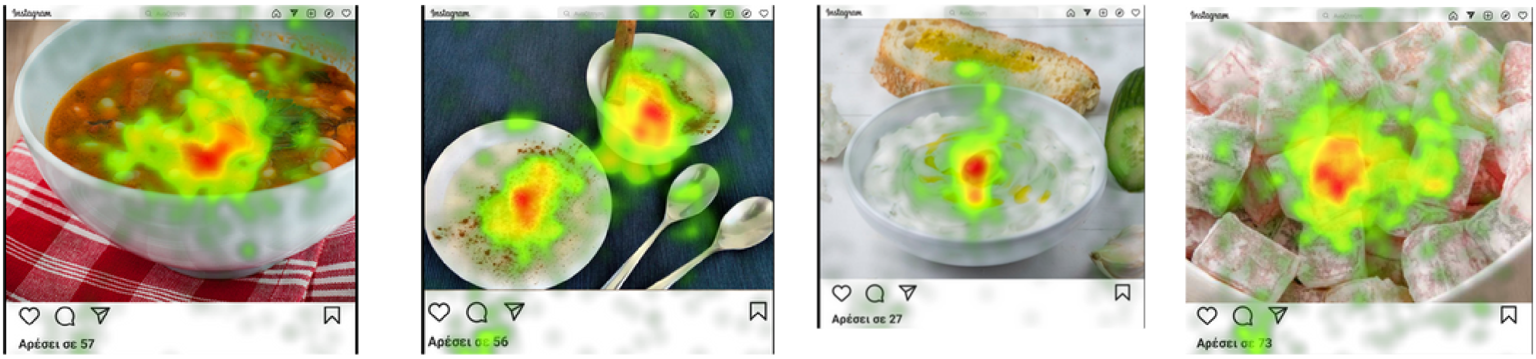

Heat map visualizations comparing participants visual attention to traditional food images: (A) high number of likes; (B) low number of likes.

(A)

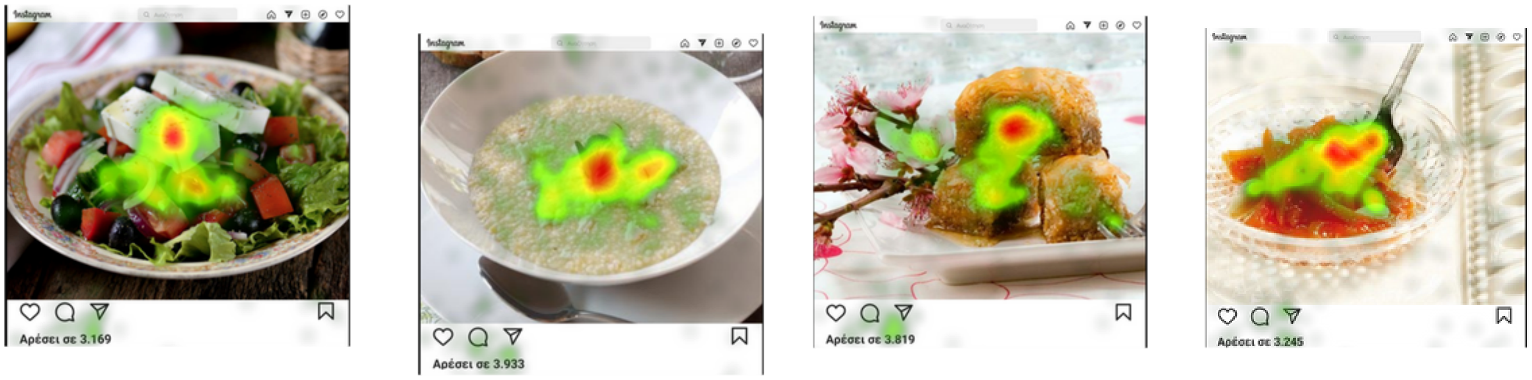

(B)

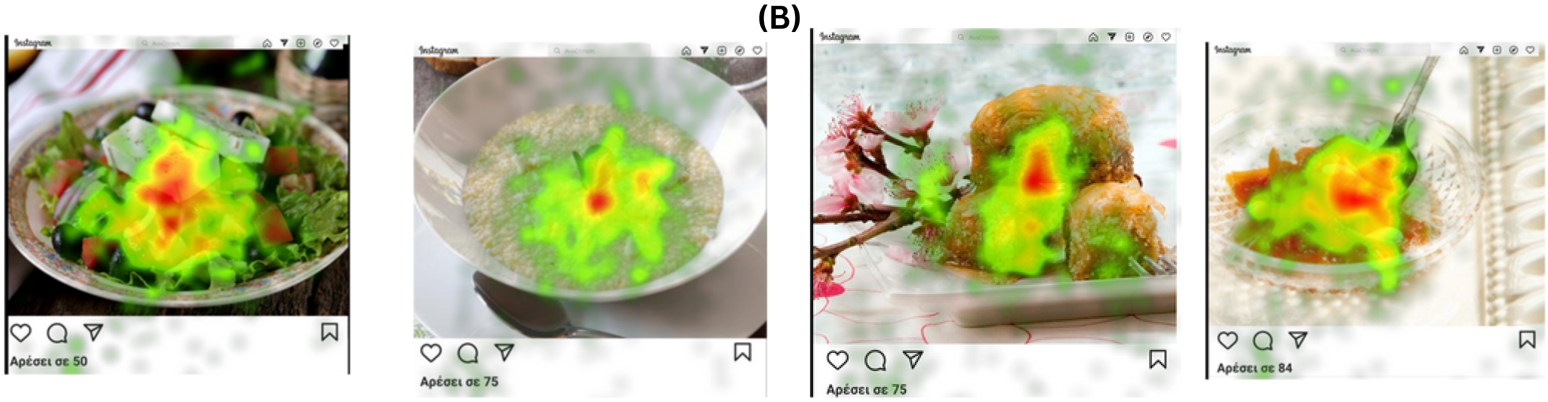

Supplement: Supplementary file 1 [file jemr-18-00062-s001.zip › jemr-3780046-supplementary.pdf]
